# Phase I trial of oxaliplatin with fluorouracil, folinic acid and concurrent radiotherapy for oesophageal cancer

**DOI:** 10.1038/sj.bjc.6604708

**Published:** 2008-10-07

**Authors:** T Conroy, F Viret, E François, J F Seitz, V Boige, M Ducreux, M Ychou, J P Metges, M Giovannini, Y Yataghene, D Peiffert

**Affiliations:** 1 Department of Medical Oncology and Radiotherapy, EA 4003, Nancy-University and Centre Alexis Vautrin, 6 avenue de Bourgogne, 54511 Vandoeuvre-lès-Nancy, France; 2 Department of Medical Oncology, Institut Paoli Calmettes, 232 boulevard Sainte Marguerite, 13009 Marseille, France; 3 Gastroenterology Unit, Centre Antoine Lacassagne, 33 avenue Valombrose, 06189 Nice, France; 4 Department of Gastroenterology, La Timone Hospital, Université de la Méditerranée, 264 rue Saint Pierre, 13005 Marseille France; 5 Gastroenterology Unit, Institut Gustave Roussy, 39 rue Camille Desmoulins, 94805 Villejuif, France; 6 Department of Medical Oncology, Val d'Aurelle Paul-Lamarque Centre, 208 rue des Apothicaires, 34298 Montpellier, France; 7 Institute of Oncology, University Hospital, 2 avenue Foch, 29200 Brest, France; 8 Oncology unit, Sanofi-Aventis France, 9 boulevard Romain Rolland, 75014 Paris, France

**Keywords:** oesophageal cancer, radiotherapy, chemotherapy, FOLFOX, oxaliplatin

## Abstract

This dose escalation study was designed to determine the maximum tolerated dose (MTD) and recommended doses (RDs) of 5-fluorouracil (5FU), folinic acid and oxaliplatin (FOLFOX) with concomitant radiotherapy in inoperable/metastatic oesophageal squamous cell carcinoma or adenocarcinoma. Patients received three courses of LV5FU2 regimen (folinic acid 200 mg m^−2^, bolus 5FU 300–400 mg/m^2^, continuous infusion 5FU 400–600 mg m^−2^ on days 1 and 2) and escalating doses of oxaliplatin 50 to 100 mg m^−2^ on day 1 (FOLFOX). This regimen was repeated every 2 weeks, concomitant to a 50-gray radiotherapy per 5 weeks. Three more cycles were delivered after completion of radiation therapy. Three to six patients were allocated to each of the five dose levels until MTD was reached. Thirty-three patients were enroled and 21 had metastatic disease. Maximum tolerated dose was oxaliplatin 100 mg m^−2^, and continuous infusion 5FU was 600 mg m^−2^ day^−^ (level 5). The most common toxicities were neutropenia, dysphagia and oesophagitis. The RDs were those of FOLFOX-4 regimen (oxaliplatin 85 mg m^−2^ and full doses of LV5FU2). The overall response was 48.5%, including 12% complete response. Response rate on primary tumour was 62.9%. This FOLFOX-4 regimen was reasonably well tolerated and effective in inoperable/metastatic oesophageal carcinoma and warrants additional investigation.

Oesophageal cancer is the eighth most common cancer worldwide, responsible for 462 000 new cases in 2002, and the sixth most common cause of death from cancer with 386 000 deaths (Parkin *et al*, 2005). Cancer of the oesophagus carries a poor prognosis with 5-year survival rates of 19.6% in the United States (Ries *et al*, 2008) and 10% in Europe (Sant *et al*, 2003). In France, the mortality rate is 90% (Bossard *et al*, 2007). According to the Burgundy cancer registry, no improvement in management of oesophageal cancer has been observed when two time periods (1976–1990 and 1997–2002) were compared. In the more recent period, only 29.6% of the patients underwent a curative resection, which yielded a 3-year survival rate of 9.7% (Bouvier *et al*, 2006).

As established by the Radiation Therapy Oncology Group (RTOG) 85-01 study, the actual standard of care for patients presenting with inoperable disease at diagnosis or with contraindications to surgery is combined chemoradiotherapy. The standard radiation dose is 50.4 gray (Gy) in 25 fractions with a cisplatin/5-fluorouracil (5FU) regimen, two cycles being administered during radiation therapy and two more cycles given after completion of the radiotherapy (Cooper *et al*, 1999). After a median of follow-up of 8 years, 22% of the patients who received combined modality therapy were alive, compared to 0% in the radiation alone arm at 3 years. However, cisplatin, especially in combination with 5FU, is known to produce significant adverse effects (Bleiberg *et al*, 1997). This was observed in the RTOG study as 20% of the patients suffered from life-threatening toxicities and 40% had to stop the chemotherapy before having completed the courses (Cooper *et al*, 1999). Safer and more efficient alternatives are therefore needed. To improve survival, synergistic combinations would be also interesting.

Oxaliplatin, a third-generation cisplatin analogue, is active in several solid tumour types, including some cisplatin/carboplatin refractory diseases. Folinic acid (FA) modulates the activity of 5FU and improves the outcome in both colorectal and gastric cancers (Machover *et al*, 1986; Johnson *et al*, 1991; Louvet *et al*, 1991; Rubin *et al*, 1996). It has been added to 5FU in several studies on oesophageal cancer with encouraging results (Highley *et al*, 1993; Stahl *et al*, 1994; Roca *et al*, 1996). The FOLFOX regimen (oxaliplatin, 5FU and FA) has already been tested in patients with recurrent or metastatic cancer of the oesophagus or cardia, and showed a significant antitumour activity along with a favourable toxicity profile (Mauer *et al*, 2005). A cohort of patients with oesophageal cancer had been treated with oxaliplatin, protracted infusional 5FU and radiotherapy in a National Cancer Institute (NCI) phase I study (Khushalani *et al*, 2002). This chemoradiotherapy regimen seemed to be effective and less toxic than cisplatin given in combination with 5FU and radiotherapy. Given these results, it is of interest to assess the tolerance and activity of chemoradiation using oxaliplatin in locally advanced and metastatic oesophageal carcinomas using a more convenient schedule. The aim of the study was to determine the dose-limiting toxicity (DLT) during chemoradiotherapy, the maximum tolerated dose (MTD), and the recommended doses (RDs) for future phase II studies.

## Patients and methods

### Patient selection

Eligible patients were required to meet all of the following criteria: locally advanced (tumour length ⩾5 cm) or metastatic adenocarcinoma, squamous cell or adenosquamous carcinoma of the oesophagus; aged 18–75 years; Eastern Cooperative Oncology Group (ECOG) performance status (PS) of 0−2; not suitable for oesophageal resection according to a multidisciplinary team; no prior chemotherapy or chest irradiation; length of radiotherapy field ⩽30 cm; peripheral neuropathy ⩽grade 1 NCI common terminology criteria (CTC) version 2.0 (NCI); sufficient calorific intake; adequate bone marrow function (absolute neutrophil count (ANC) ⩾2 × 10^9^ l^−1^, platelet count ⩾100 × 10^9^ l^−1^), normal renal and liver function. Patients were excluded if they were presenting small cell or undifferentiated carcinoma of the oesophagus, complete dysphagia, weight loss >20% of normal body weight, history of prior malignancies (other than cured non-melanoma skin cancer, cured cervical carcinoma *in situ* or stage I or II node-negative head-and neck cancer cured more than 3 years ago), prior neck radiotherapy with field overlapping the proposed oesophageal radiotherapy field, brain or leptomeningeal metastases, tracheo–oesophageal fistula or biopsy-proven invasion of the tracheo–bronchial tree. The study was designed according to the Committee for Proprietary Medicinal Products (CPMP) guideline for anticancer therapy (EMEA, 2003), and conducted in accordance with the Declaration of Helsinki (Declaration of Helsinki), Good Clinical Practice guidelines and applicable local legal requirements. The protocol was approved by the Ethical Committee of Lorraine. Written informed consent was obtained from all patients.

### Pretreatment evaluation

Screening assessments consisted of clinical history, recording of concomitant medications, physical examination, ECOG performance status, haematological and biochemical parameters and electrocardiogram. Disease extension was assessed by oesophagoscopy and biopsies, chest radiography, barium oesophagram, chest and abdominal computed tomography (CT) and transoesophageal ultrasonography (if possible).

### Radiotherapy

External beam radiation therapy was delivered by linear accelerator using an energy >6 MV. Three or four beams were used, according to the dosimetry. All fields were treated each day. A total dose of 50 Gy in 25 fractions was prescribed at the ICRU reference point, delivered 5 days a week. For the first plan, 40 Gy was delivered to the PTV, defined as the GTV with a 5-cm margin in the cranio–caudal direction and 3 cm radially, using custom blocks. The primary tumour and regional lymph nodes were included in this initial volume. A 10-Gy boost was then delivered to a reduced volume (primary tumour and nodes with a 1-cm margin). The maximum dose to the spinal cord was 40 Gy. Portal images for each field were performed at the initiation and at the completion of radiotherapy.

### Chemotherapy and study design

Three FOLFOX cycles were administered every 2 weeks during the 5 weeks of the radiotherapy course. Then, in the absence of tumour progression and/or limiting toxicity, three more cycles were also administered. Metastatic patients who had stable disease or objective response after radiotherapy were to continue to receive FOLFOX every 2 weeks until limiting toxicity, lack of clinical benefit, refusal or disease progression. Patients received the following medications during each chemotherapy cycle: oxaliplatin X mg m^−2^ as a 2 h i.v. infusion, on day 1; FA 200 mg m^−2^ i.v. infusion over 2 h (concomitantly to oxaliplatin on day 1 and alone on day 2); 5FU bolus Y mg m^−2^ day^−1^ 10 min i.v. bolus, following FA administration on days 1 and 2; 5FU Z mg m^−2^ day^−1^ 22 h i.v. continuous infusion, following 5FU bolus administration on days 1 and 2. The dose levels of the escalation design are described in Table 1. Oxaliplatin dose was reduced of one level in case of grade 3 neutropenia with fever and/or infection or grade 4 neutropenia, in case of grade 3–4 thrombopenia or grade 2 neurotoxicity. The 5FU bolus was not administered in the event of a grade 3–4 diarrhoea or mucositis/oesophagitis.

Three to six patients were allocated to each dose level until MTD was reached. Three patients at a given dose level were to complete the radiation therapy before patients were included at next dose level. The MTD was defined as the dose level at which at least three out of three or six patients experienced a DLT. If at least three out of six patients had DLT at a given dose level, six patients were included at the previous dose level. Each dose escalation was discussed between the investigators, coordinator and sponsor. The RD level was the dose level where two or less patients out of six had a DLT.

### Endpoints

The primary objective of the study was to document the MTD of dose-escalated FOLFOX, when given with concomitant radiotherapy for inoperable advanced or metastatic oesophageal cancer treatment. The DLT was defined as any of the following event occurring during the concomitant chemoradiotherapy period; an NCI-CTC grade 4 thrombocytopenia, a febrile grade 4 neutropenia, a grade 4 leucopoenia, a neutropenia leading to >7 days of interruption of radiotherapy, or any grade 3–4 non-haematological toxicity attributed to concomitant chemoradiotherapy (except oesophagitis, dysphagia, nausea and vomiting). Patients must have received a minimum of three cycles of chemotherapy and 40 Gy over a maximum of 6 weeks to be considered evaluable for response, unless early disease progression occurred.

The secondary objectives were to determine the tumour response rates using the World Health Organisation (WHO) criteria, the duration of response for complete or partial responders (CR or PR), the progression-free survival (PFS), the overall survival and the degree of relief of dysphagia. Patients were restaged 8 weeks after the completion of radiotherapy, and then, in patients achieving a CR every 3 months until progression. All lesions (primary tumour, involved nodes and metastases) had to be assessed with CT scan, barium esophagram and endoscopy. Patients with metastatic disease had a tumour evaluation after every four cycles of chemotherapy. All objective responses were confirmed by a second evaluation after 4 weeks. The duration of response was defined as the time from the beginning of treatment (for PR) or from the time of CR documentation to the time of the documented progression. Toxicity was graded weekly during the whole treatment (concomitant chemoradiotherapy and subsequent chemotherapy) according to the NCI-CTC version 2.0 (NCI) and specific neurotoxicity scale. All patients who received at least one dose of chemotherapy were considered as evaluable for safety. Dysphagia was graded at each visit to assess symptomatic relief. Progression-free survival was calculated from the start of treatment until death, cutoff date or date of the last follow-up visit. Overall survival was measured from the initiation of the treatment to the time of death, the date of the last follow-up visit or the cutoff date.

### Statistical analysis

The primary efficacy endpoint and response rates were assessed based on both the intent-to-treat (ITT) and the evaluable per protocol (PP) populations. All other endpoints were performed on the ITT population. PFS and overall survival were estimated using the Kaplan–Meier method.

### Follow-up

During radiotherapy, symptoms, physical examination and haematologic parameters were recorded weekly. In the case of grade 3–4 neutropenia or thrombocytopenia, complete blood counts were performed twice a week until recovery to ⩽grade 2. The clinical follow-up (as described above) was then performed every 2 weeks with complete blood counts assessed weekly and up to 8 weeks after completion of radiation therapy. Patients were restaged 8 weeks after completion of radiotherapy and were then observed every 3 months. Dysphagia grade was assessed 4 weeks after the end of radiotherapy, and thereafter every 3 months until disease progression using NCI-CTC criteria.

## Results

### Patient characteristics

**Figure 1:**
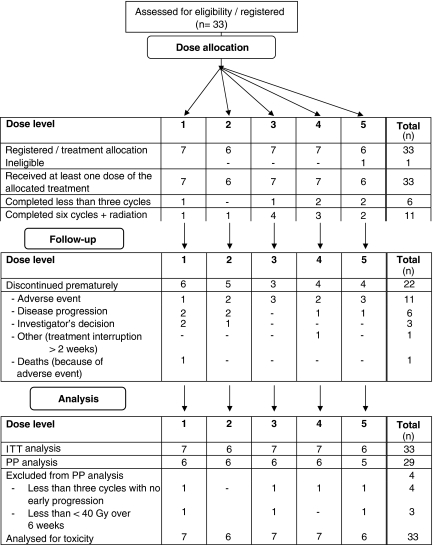
Diagram of the study. ITT=intent-to-treat; PP=per protocol.

Between March 2000 and October 2004, 33 patients were enroled in seven French institutions. One patient included in the level 5 cohort was subsequently considered as ineligible for DLT analysis because of the baseline tracheal invasion. However, he remained assessable for safety. He was not replaced as the MTD had already been reached. The safety and ITT sets included 33 patients, and the PP set 29 patients (Figure 1). Main baseline characteristics are listed in Table 2.

### Treatment delivered

Overall, the median number of cycles received was 6 (range: 1–10 cycles), with 19 patients (57.6%) having received ⩾6 cycles. Dose intensity, relative dose intensity, dose at first cycle and dose at last cycle data were all similar among all the dose levels, for both oxaliplatin and 5FU. Twenty patients (60.6%) had a treatment delay (only one cycle in 16 cases). Of the 31 patients who received at least two cycles, six patients (19.4%) had an oxaliplatin dose reduction, six (19.4%) patients had a single 5FU dose reduction and one patient had two 5FU dose reductions. The main reasons for either cycle delays or dose reductions were the apparition of haematological toxicities.

### Maximum tolerated dose and dose-limiting toxicities

The number of patients who experienced DLTs and the type of DLTs are provided in Table 3. The MTD was reached at dose level 5, where three out of five patients experienced DLTs (grade 3 asthenia, grade 4 asthenia and grade 3 diarrhoea). The majority of DLTs was of grade 3, occurring mainly at cycle 3. All but one DLT (a grade 4 febrile neutropenia) were grade 3–4 non-haematological toxicities. The main observed toxicities at each level for concomitant chemoradiotherapy and all cycles of chemotherapy are summarised in Table 4.

### Efficacy

**Figure 2:**
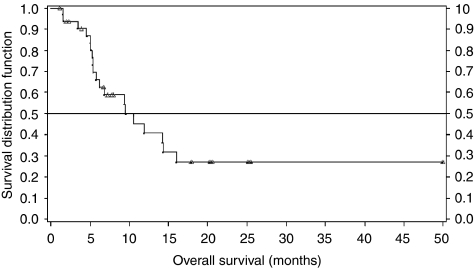
Overall survival. Censored values are presented with a triangle.

Tumour responses are described in Table 5. Sixteen (48.5%; 95% CI: 30.8–66.5) patients showed a response to the treatment (CR or PR), with four (12.1%) patients having a CR. The overall response rate (among the 26 assessable patients) was 61.5%. Out of the 12 patients with locally advanced disease, 3 CR and 5 PR (66.6%) were observed. None of these patients had secondary surgery. Primary tumour response to chemoradiotherapy was assessed in 27 patients. Objective responses were observed in 17 patients (62.9%), including six patients (22.2%) with CR. For all patients, the median duration of response was 11.8 months (95% CI: (9.0; –)) and median PFS was 6.7 months (95% CI: (4.3; 24.1)). Figure 2 presents the overall survival. Median survival was 9.5 months (95% CI: (5.8; 16.0)). At 15 months, five patients with metastatic disease and seven patients with locally advanced tumour were still alive.

### Adverse events

Seven (21.8%) patients died during the treatment period. One patient died from toxicities considered as related to study treatment (level 2). This patient had a medical history of heavy smoking, coronary insufficiency and obesity. He had metastases in the lung and bilateral paratracheal metastatic nodes. Six weeks after the sixth course of chemotherapy, the patient experienced acute respiratory failure and the chest X-rays showed diffuse alveolo–interstitial pneumopathy, leading to his death. The investigator considered that the death might be related to a delayed pulmonary toxicity because of radiotherapy.

Oesophagitis was the most commonly experienced serious adverse events (six patients; 18.2%). Nineteen patients experienced toxicities that led to definitive or temporary treatment discontinuation. The most common toxicities that led to treatment discontinuation were asthenia (five patients) and oesophagitis (four patients). Fifteen patients ceased the treatment because of a toxicity considered to be related to the experimental chemotherapy regimen (relationship likely or unknown). One patient developed an acquired oesotracheal fistula at the upper pole of an oesophageal prosthesis after the eighth course of chemotherapy. The investigator considered that the fistula was related to a traumatism because of oesophageal prosthesis.

No obvious differences between dose levels were observed with regard to the frequency of deaths, other serious adverse events and discontinuations related to adverse events.

### Assessment of dysphagia

Four weeks after the completion of concomitant chemoradiotherapy, dysphagia was described in 19 patients (59.4%), with symptom improving with time. At 15 months, no residual dysphagia was observed in the surviving patients.

## Discussion

The primary objective of this phase I study was to document the MTD and RDs of a dose-escalated combination therapy containing FOLFOX with concomitant radiotherapy as a first-line treatment for inoperable advanced or metastatic oesophageal cancer. Maximum tolerated dose was reached at dose level 5, where three out of five patients experienced DLTs. The RD was therefore dose level 4 (85 mg m^−2^ i.v. oxaliplatin, 200 mg m^−2^ i.v. FA, 400 mg m^−2^ day^−1^ bolus 5FU and 600 mg m^−2^ day^−1^ 22-h infused 5FU), which is a full-dose FOLFOX 4 regimen (de Gramont *et al*, 2000). A major advantage of this treatment is that it can be safely given on an outpatient basis. Approximately half of the patients (48.5%) had an objective tumour response with associated relief of dysphagia. Response rate appears to be higher on the primary tumour site (62.9%). The lower median survival time in this study, when compared to the other chemoradiation studies (Cooper *et al*, 1999; Minsky *et al*, 2002), most probably reflects the choice of our inclusion criteria (metastatic disease or inoperable tumour ⩾5 cm). The incidence of dose reduction because of toxicity was low for both oxaliplatin and 5FU. Grade 3–4 haematological toxicities were rare, for both oxaliplatin and 5FU and decreased as the study progressed, indicating that these toxicities did not worsen with continued administration of the experimental treatment.

A prior phase I study combined oxaliplatin, protracted infusion 5FU and radiation in a preoperative setting (Khushalani *et al*, 2002). Forty patients received oxaliplatin (dose range: 85–100 mg m^−2^) on days 1, 15 and 29 and continuous infusion 5FU (dose range: 160–200 mg m^−2^ day^−1^) from days 8–42, concurrently with a total radiotherapy dose of 50.4 Gy. Most of the patients (85%) had adenocarcinoma. Thirteen (32/5%) patients underwent curative surgery. A pathologic complete response (pCR) rate of 38% was observed. Therefeore, the RD was oxaliplatin 85 mg m^−2^, protracted infusion 5FU 180 mg m^−2^ along with radiotherapy 50.4 Gy in 28 fractions of 1.86 Gy per fraction. A retrospective series of 42 patients who received this regimen observed a 25% pCR rate out of 20 patients undergoing surgery (O'Connor *et al*, 2007). There was only one CR out of 13 patients treated with definitive chemoradiation. The Southwest Oncology Group phase II study in potentially curable stage II or III oesophageal adenocarcinoma is currently ongoing. Another phase I trial was conducted, using the combination of oxaliplatin, cisplatin and protracted infusion 5FU associated with a standard radiotherapy dose of 50.4 Gy (Maurel *et al*, 2005). Nineteen patients were treated and two pCR were observed in 12 patients who underwent surgery. Toxicity was mild and a prospective phase II study was planned for resectable oesophageal and gastric cancers.

The combination of oxaliplatin and capecitabine has recently been tested in patients with advanced or metastatic oesophageal cancer, mainly adenocarcinoma (88%). The response rate was 39% and the median survival time was 8 months (van Meerten *et al*, 2007). In the phase III trial, REAL 2 comparing oxaliplatin to cisplatin and capecitabine to 5FU, 964 eligible patients with advanced oesophageal or gastric cancers were randomised (Cunningham *et al*, 2006). Oesophageal and oesophagogastric junction tumours represented 34.5 and 25.7% of the primaries. The authors conclude that capecitabine may replace 5FU and oxaliplatin may replace cisplatin for the treatment of advanced oesophagogastric cancer.

This study suggests that further investigations are required in a phase II study using the recommended dose level 4, the FOLFOX 4 regimen. Such a phase II–III randomised study is ongoing; it compares chemoradiotherapy with FOLFOX 4 regimen (ie, dose level 4 of this study) *vs* chemoradiotherapy with 5FU/cisplatin (RTOG regimen) as a first-line treatment for patients with inoperable oesophageal cancer. In conclusion, oxaliplatin, 5FU and FA with concomitant radiotherapy (50 Gy) is feasible and effective in patients with inoperable locally advanced or metastatic oesophageal cancer.

## Figures and Tables

**Table 1:** FOLFOX (5-fluorouracil (5FU), folinic acid and oxaliplatin) dose levels

**Dose level^a^**	**Oxaliplatin X mg m^−2^**	**Bolus 5FU Y mg m^−2^ day^−1^**	**Continuous infusion 5FU Z mg m^−2^ day^−1^**
1	50	300	400
2	50	400	600
3	75	400	600
4	85	400	600
5	100	400	600

a All dose levels also included a 200 mg m^**−**2^ folinic acid i.v. infusion.

**Table 2:** Patient characteristics (ITT population)

**Total (*n*)**	**33**
*Age (years)*	
Mean (s.d.)	58.2 (9.20)
Range	37–75
	
*Gender (*n, %)	
Male	26 (78.8)
	
*ECOG performance status (*n, %)	
0	5 (14.7%)
1	27 (79.4%)
2	2 (5.9%)
	
*Stage (*n, %)	
III	12 (36.4)
IV	21 (63.4)
	
*Dysphagia grade (*n, %)	
1	9 (27.2)
2	20 (60.6)
3	4 (12.1)
	
*Histologic type (*n, %)	
Adenocarcinoma	10 (30.3)
Squamous cell	20 (60.6)
Mixed	3 (9.1)
	
*Site of distant metastases (*n=*21;* n, %)	
Lung	6 (28.5)
Lymph nodes	20 (95.2)
Liver	7 (33.3)
Peritoneum	1 (4.7)

Stage III=T3 N1 M0 or T4 N0 or 1 M0; Stage IV=any T any N M1.

**Table 3:** Number of patients with dose-limiting toxicity (DLT) by dose level

**Dose level**	**1 (*n*=6)**	**2 (*n*=6)**	**3 (*n*=6)**	**4 (*n*=7)**	**5 (*n*=5)**	**Combined (*n*=30)**
Number of patients with DLT	2	1	2	1	3	9
Number of DLTs	3	2	3	1	3	12
						
*Type of DLT*						
Anorexia	1	1	1	1	—	4
Asthenia	1	—	1	—	2	4
Epithelitis	1	—	—	—	—	1
Febrile neutropenia	—	—	1	—	—	1
Infection	—	1	—	—	—	1
Diarrhoea	—	—	—	—	1	1

**Table 4:** Grade 3–4 toxicities at various dose levels of oxaliplatin and 5FU (all cycles)

**Dose level**	**1 (*n*=7)**	**2 (*n*=6)**	**3 (*n*=7)**	**4 (*n*=7)**	**5 (*n*=6)**	**Combined (*n*=33)**
*Toxicity (NCI-CTC;* n)						
Haemoglobin	—	1	1	—	3	5
WBC	1	1	5	1	—	8
Granulocytes	1	2	5	1	—	9
Platelets	—	—	1	1	1	3
Febrile neutropenia	—	1	1	—	—	2
Anorexia	1	1	2	1	2	7
Asthenia	1	—	2	1	3	7
Cough	—	—	—	—	2	2
Dehydration	1	—	—	1	—	2
Diarrhoea	—	—	—	1	1	2
Dysphagia	3	2	4	3	2	14
Dyspnoea	—	—	1	—	1	2
Haemorrhage	1	1	—	—	—	2
Mucositis	—	1	1	—	—	2
Nausea	1	—	—	1	—	2
Vomiting	1	—	—	1	—	2
Oesophagitis	2	1	2	2	—	7
Pain	—	—	1	—	1	2
Septic shock	—	—	1	1	—	2
Neurotoxicity	—	1	—	—	—	1

**Table 5:** Tumour responses on all disease sites

**Dose level**	**1 (*n*=7)**	**2 (*n*=6)**	**3 (*n*=7)**	**4 (*n*=7)**	**5 (*n*=6)**	**Combined (*n*=33)**
*Response (*n)						
Not evaluable	3	—	1	2	1	7
Complete response	2	—	2	—	—	4
Partial response	—	4	3	3	2	12
Stable disease	—	1	1	1	—	3
Progression	2	1		1	3	7
Number of responses	2	4	5	3	2	16
